# T248. PSYCHOPATHOLOGY IN F2X.X-UNAFFECTED CO-TWINS AS A VULNERABILITY INDICATOR OF PSYCHOSIS

**DOI:** 10.1093/schbul/sby016.524

**Published:** 2018-04-01

**Authors:** Rikke Hilker, Mette Nielsen, Christian Legind, Maria H Jensen, Simon Anhøj, Brian Broberg, Birgitte Fagerlund, Merete Nordentoft, Birte Glenthøj

**Affiliations:** 1 Mental Health Center Glostrup; 2 Center for Neuropsychiatric Schizophrenia Research (CNSR); 3 Center for Neuropsychiatric Schizophrenia Research (CNSR), Center for Clinical Intervention and Neuropsychiatric Schizophrenia Research (CINS), Mental Health Centre Glostrup, University of Copenhagen; 4 Center for Neuropsychiatric Schizophrenia Research (CNSR), Center for Clinical Intervention and Neuropsychiatric Schizophrenia Research (CINS), Copenhagen University Hospital; 6 University of Copenhagen

## Abstract

**Background:**

Studies have shown that the risk of developing schizophrenia is associated with an increased risk of most other psychiatric disorders1 and that the familial transmission of risk extends across diagnostic categories.2 In twin studies, unaffected twins may not be completely free of symptomatology even when they do not fulfill diagnostic criteria for a psychiatric illness, but this has not been systematically tested in a twin design.

The aim of the study was to investigate subtle psychopathology in unaffected co-twins from proband twin pairs, where one twin had a schizophrenia spectrum disorder diagnosis (ICD-10: F2x.x), and compare the level of psychopathology of F2x.x-unaffected co-twins to that of healthy twins.

**Methods:**

We conducted a multimodal, cross-sectional combined clinical and register-based nation-wide twin study, by including twin pairs where one or both twins had a diagnosis in the schizophrenia spectrum (identified by linking The Danish Twin Register and the Danish Psychiatric Central Research Register). A group of age-and gender matched healthy twin pairs were included. All subjects underwent ratings of psychopathology with Positive and Negative Syndrome Scale (PANSS), Comprehensive Assessment of At Risk Mental State (CAARMS), and Clinical Global Impression (CGI). The current level of functioning was estimated with Global Assessment of Function (GAF).

**Results:**

A total of 219 twins were included; i.e. proband twins (n=65), F2x.x-unaffected co-twins (n=56) and healthy twins n=98). For unaffected co-twins, the mean PANSS total score was 38 (SD 12.6), the CGI score was 1.7 (SD 1.1) and the GAF score was 74 (SD 13.3), which were all significantly higher than in healthy twins (all p<0.02), who had a mean PANSS total score of 32 (SD 5.7), a CGI score of 1 (SD 0.3) and a GAF score of 85 (SD 7.3). For CAARMS, the following items were significantly more severe in the unaffected co-twins compared to healthy twins: anxiety (p=0,021), OCD (p=0.008), stress tolerance (p=0.001), aggression (p=0.01), inappropriate affect (p=0.03), social isolation (p=0.01), and impaired role-function (p=0.001).

**Discussion:**

These preliminary results indicate a subtle but significant level of psychopathology in unaffected co-twins of probands affected with a schizophrenia spectrum disorder compared to healthy twins, measured by PANSS and CGI. Results from CAARMS indicate specific areas of interest, clustered around emotional and behavioral symptoms like anxiety, stress intolerance, social isolation, inappropriate affect/aggression and impaired role-function. This may suggest subtle symptomatology in the f2x.x-unaffected co-twins, which may contribute to the significantly lower level of function in the co-twins compared to healthy twins. The heritability of these measures of psychopathology will be examined using Structural Equation Modelling on the whole cohort of probands, unaffected co-twins and healthy twins.

**References:**

1. Mortensen PB, Pedersen MG, Pedersen CB. Psychiatric family history and schizophrenia risk in Denmark: which mental disorders are relevant? Psychol Med 2010; 40: 201–10.

2. Rasic D, Hajek T, Alda M, Uher R. Risk of mental illness in offspring of parents with schizophrenia, bipolar disorder, and major depressive disorder: a meta-analysis of family high-risk studies. Schizophr Bull 2014; 40: 28–38.

